# Classification of LRS Bianchi-I spacetime in context of *f*(*T*) gravity via its self-similar symmetry

**DOI:** 10.1371/journal.pone.0334004

**Published:** 2025-12-05

**Authors:** Rabeb Sidaoui, A. H. A. Alfedeel, Jamshed Khan, Mohammed Alsharafi, E. I. Hassan, Khaled Aldwoah

**Affiliations:** 1 Department of Mathematics, College of Science, University of Ha’il, Ha’il, Saudi Arabia; 2 Department of Mathematics and Statistics, Imam Mohammad Ibn Saud Islamic University (IMSIU), Riyadh, Saudi Arabia; 3 Government Post Graduate College Lakki Marwat, Khyber Pakhtunkhwa, Pakistan; 4 Department of Mathematics, Faculty of Science, Sana’a University, Sana’a, Yemen; 5 Department of Mathematics, Faculty of Science, Islamic University of Madinah, Madinah, Saudi Arabia; Lahore School of Economics, PAKISTAN

## Abstract

This study investigates self-similar vector fields of locally rotationally symmetric Bianchi type–I spacetimes within the framework of *f*(*T*) gravity, incorporating a perfect fluid as the matter source. The analysis demonstrates that certain spacetimes with a perfect fluid admit self-similar vector fields of infinite, first, zeroth, and second kinds. To address this problem, the Rif tree approach has been employed. In this method, the symmetry and field equations are transformed using Maple, which generates a set of constraints on the spacetime functions. These constraints are then applied to solve the symmetry equations, ultimately yielding the exact form of the self-similar vector field. Furthermore, the physical quantities—energy density *ρ*, pressure *p*, torsion scalar *T*, and torsion-based function *f*(*T*)—are calculated for each solution, providing a comprehensive understanding of the physical and geometric properties of the spacetime. In addition, the kinematic variables associated with the derived metrics have also been calculated. The findings of this study have significant applications in cosmology, astrophysics, and modified gravity theories, particularly in modeling cosmic evolution, black hole formation, and anisotropic spacetime structures. The classified self-similar solutions in *f*(*T*) gravity contribute to understanding gravitational collapse, the dynamics of the early universe, and the stability of astrophysical objects.

## Introduction

Einstein’s field equations (EFEs) provide a relationship between the physical and geometrical behavior of our universe. EFEs are written as [1]:

$$G_{ij}=kT_{ij}\;(i,j=0,1,2,3),$$ (1)

where the term $G_{ij}=R_{ij}\;-\;\frac{37}{2}Rg_{ij}$ represents the Einstein tensor, which describes the geometrical (curvature) behavior of the universe, and *k* denotes a constant. Moreover, *R*_*ij*_ represents the Ricci tensor, *g*_*ij*_ the metric tensor, *R* the Ricci scalar, and *T*_*ij*_ the energy-momentum tensor, which explains the physical behavior of the universe.

Eq (1) represents a set of partial and nonlinear differential equations. Due to these complexities, obtaining the general solution of EFEs is challenging. To simplify the system (1), certain assumptions are required, one of the most common being symmetry restrictions. These symmetries are categorized based on their ability to preserve various physical aspects such as the Ricci scalar, metric tensor, geodesics, and curvature, among others. The role of these symmetries is to provide exact solutions to (1) and to classify existing solutions. Here, the focus is on symmetries, including self-similar symmetry, homothetic, conformal, and Killing symmetry, which preserve the metric tensor.

For more details on homothetic, conformal, and Killing symmetries, one can refer to [2–9].

As this article focuses on self-similar symmetry, also called self-similar vector fields (SSVFs), which prove to be useful in simplifying EFEs, a vector field *V* is known as an SSVF when it satisfies the following two conditions [10,11]:

$${ℒ}_{V}u_{i}=a_{1}u_{i},\;{ℒ}_{V}h_{ij}=2a_{2}h_{ij}.$$ (2)

Here, ℒ denotes the Lie derivative operator, *a*_1_ and *a*_2_ are constants, $h_{ij}=g_{ij}+u_{i}u_{j}$ is the projection tensor, and *u*_*i*_ represents the four-velocity vector. This symmetry reduces the partial nature of Eq (1) into an ordinary differential form, which is comparatively easier to handle. By imposing specific conditions on *a*_1_ and *a*_2_, SSVFs are classified into various types as follows [12]:

(i) First kind (or homothetic vector fields), if $a_{1}=a_{2}$.
(ii) Zeroth kind, when $a_{1}=0,\;a_{2}\neq 0$.
(iii) Infinite kind, if $a_{1}\neq 0,\;a_{2}=0$.
(iv) Second kind, when $a_{2}\neq a_{1}\neq 0,1$.

Furthermore, if $a_{1}=a_{2}=0$, then SSVFs correspond to Killing vector fields.

This symmetry plays a significant role in describing various phenomena, such as gravitational collapse, asymptotic properties of certain general models, critical phenomena, and black hole mass determination [10,13]. Additionally, its applications extend to cosmic censorship, cosmological perturbations, the investigation of cosmological voids, the study of primordial black holes, star formation, and astrophysics [12,14].

Given the importance of this symmetry, researchers in general relativity have extensively studied its implications. The classification of locally rotationally symmetric spacetimes based on their self-similar symmetry was conducted by Sharif and Amir [15], where they obtained infinite, second, first, and zeroth kinds of SSVFs for the said spacetimes. Cylindrical and spherically symmetric self-similar solutions were explored by Sharif and Aziz [16,17]. The study of SSVFs for plane symmetric spacetimes was presented by Shabbir and Khan [18], where all kinds of SSVFs were discussed. For static symmetric (spherically and axially symmetric) spacetimes, SSVFs were explored by the same authors [19,20], identifying all types of SSVFs for these spacetimes. For the Kantowski-Sachs metric, self-similar solutions were studied by Gad et al. [21].

Although Einstein’s General Relativity (GR) has been remarkably successful in describing gravitational phenomena on various scales, it faces significant challenges in explaining certain observations of our universe. For instance, the accelerated expansion of the cosmos, the nature of dark energy and dark matter, and the behavior of gravity in strong-field regimes remain inadequately addressed within the framework of GR. These limitations motivate the development of modified theories of gravity, such as *f*(*R*) [22–25], *f*(*T*), or *f*(*R*,*T*) [26–29] theories, which aim to provide a more complete and consistent explanation of cosmological and astrophysical phenomena without invoking exotic components. These modifications offer a promising pathway toward understanding the true nature of gravity and the large-scale structure of the universe.

*f*(*T*) gravity is considered one of the most pivotal and widely studied extensions of gravitational theories in modern theoretical physics. This theory plays a crucial role in explaining the accelerated expansion of the universe. By analyzing viable classes of *f*(*T*) gravitational models, researchers can explore transition redshifts, dynamic phenomena, and other cosmological features [30–32]. This theory has been instrumental in understanding large-scale cosmic processes, significantly contributing to the development of modern cosmological models [33]. Additionally, *f*(*T*) gravity aligns remarkably well with observational data from binary pulsars and the solar system [34]. It also provides a consistent framework for understanding inflation, offering a well-structured alternative to traditional approaches [35,36]. In *f*(*T*) gravity, various functional forms have been proposed to explain cosmic acceleration. The simplest is the linear form f(T)=T, which reduces to general relativity in the teleparallel framework. Power-law models $f(T)=\mu T^{n}$ have been explored to describe different cosmic epochs, while exponential forms $f(T)=Te^{\lambda T}$ address specific observational features. Logarithmic models f(T)=αlnT can produce deceleration-acceleration transitions, and polynomial forms like $f(T)=\beta T+mT^{2}$ provide flexibility in matching observations [37].

The significance of *f*(*T*) gravity depends on specific field equations derived from the theory. Although these field equations are second-order, solving them still requires advanced analytical techniques. In Ref. [38], the authors explored multiple solutions within this theory, including charged gravastars, under the framework of conformal symmetry in spacetime. This symmetry plays a crucial role in *f*(*T*) gravity, helping to derive solutions for compact stellar objects [39]. These studies highlight the importance of *f*(*T*) gravity as a theoretical framework.

Bianchi models are among the most important in cosmology, providing better explanations for cosmic expansion. Consequently, researchers have shown considerable interest in these models across various gravity theories. The study of the LRS Bianchi type I metric in teleparallel gravity was conducted by Sharif and Jabbar [40]. The same spacetime was classified according to its Noether symmetries by Malik et al. [41] in f(R,φ,χ) theory. The study of LRS Bianchi type I metrics in *f*(*R*,*T*) theory is presented in [42,43].

For solving the corresponding symmetry equations, researchers have traditionally employed the direct integration approach in all aforementioned classifications. However, this approach is often cumbersome, time-consuming, and may overlook significant metrics. To overcome these challenges and ensure comprehensive classification, recent studies in general relativity have increasingly relied on computer algorithms.

The computer-based approach was initially introduced by Reid et al. [44] using the Maple package rifsimp, later refined by Wittkopf [45]. Recent studies employing the Rif tree approach have explored symmetries in various spacetimes, including Killing symmetries in static cylindrically symmetric configurations [46] and self-similar vector fields within Bianchi type III spacetime [47]. This approach significantly reduces complexity and provides essential insights into the system, such as the number of existing solutions, even without explicitly solving it. The command caseplot is used to graphically represent the Rif algorithm, generating a Rif tree. The nodes of this tree correspond to pivots containing the highest-order derivative functions present in the system. For the Rif algorithm to produce a meaningful Rif tree, the operational issue of variable ordering must be considered, as different variable orderings can yield different trees. Since no general theory dictates optimal variable ordering, trial and error remains the primary approach.

The utilization of the Rif tree approach in this study aims to identify all potential metrics possessing the required symmetry. These identified metrics are then used to solve the desired symmetry differential equations. The process begins by developing a computer algorithm that simplifies the equations and generates the Rif tree, containing metric function conditions that are subsequently used to solve the SSVF equations.

Inspired by previous literature, this paper aims to classify the LRS Bianchi type I spacetime through its SSVFs within the framework of *f*(*T*) gravity, adopting the Rif tree approach.

In the succeeding sections of this study, we will intend to proceed with the six main objectives that are derivation of symmetry equations, derivation of field equation of *f*(*T*) theory, presenting main results, study of kinematic variables and summary of the whole study.

## 1 Symmetry equations

The standard representation of the LRS Bianchi-I spacetime is given as [1]:

$$ds^{2}=-dt^{2}+K_{1}^{2}(t)dx^{2}+K_{2}^{2}(t)(dy^{2}+dz^{2}).$$ (3)

Here, *K*_1_(*t*) and *K*_2_(*t*) cannot vanish. The set {$\partial_{x},\partial_{y},\partial_{z},z\partial_{y}\;-\;y\partial_{z}$} represents the minimum Killing vector fields (KVFs) admitted by (3).

For this spacetime, we have *u*_*i*_ = (1,0,0,0). Using this *u*_*i*_ and the SSVF Eq (2) in the metric (3), we obtain the following set of equations:

$$V_{,x}^{0}=V_{,y}^{0}=V_{,z}^{0}=0,$$ (4)

$$V_{,t}^{0}=a_{1},$$ (5)

$$V_{,t}^{1}=V_{,t}^{2}=V_{,t}^{3}=0,$$ (6)

$$K_{1}^{\prime }V^{0}+K_{1}V_{,x}^{1}=a_{2}K_{1},$$ (7)

$$K_{1}^{2}V_{,y}^{1}+K_{2}^{2}\;V_{,x}^{2}=0,$$ (8)

$$K_{1}^{2}V_{,z}^{1}+K_{2}^{2}\;V_{,x}^{3}=0,$$ (9)

$$K_{2}^{\prime }V^{0}+K_{2}V_{,y}^{2}=a_{2}K_{2},$$ (10)

$$V_{,z}^{2}+V_{,y}^{3}=0,$$ (11)

$$K_{2}^{\prime }V^{0}+K_{2}V_{,z}^{3}=a_{2}K_{2}.$$ (12)

Eq (4) clearly indicates that *V*^0^ is independent of *x*,*y*,*z*, and thus we obtain:


$$V^{0}=a_{1}t+\text{constant}.$$

Furthermore, from Eq (6), it is evident that *V^i^* for i=1,2,3 are independent of the variable *t*.

## 2 Field equations

In this section, the derivation and solution of field equations along with the above SSVFs equations will be carried out for the spacetimes under study in the frame work of *f*(*T*) gravity. This gravity is built upon the Weitzenböck connection, where the geometry, in conjunction with the metric, is characterized by:


$$ds^{2}=g_{ij}dx^{i}dx^{j},$$

where $g_{ij}=\eta_{mn}e_{i}^{m}e_{j}^{n}$ with $\eta_{mn}=\text{diag}(-1,1,1,1)$ denote metric components, while $e_{i}^{m}$ defines the tetrad field. The Weitzenböck connection is constructed mathematically through the tetrad field as:


$$\Gamma_{\alpha \beta }^{\delta }=e_{i}^{\delta }\partial_{\beta }e_{\alpha }^{i}=-e_{\alpha }^{i}\partial_{\beta }e_{i}^{\alpha }.$$

The torsion tensor is constructed from the antisymmetric part of this connection as:


$$T_{\alpha \beta }^{\delta }=\Gamma_{\beta \alpha }^{\delta }-\Gamma_{\alpha \beta }^{\delta }=e_{i}^{\delta }(\partial_{\alpha }e_{\beta }^{i}-\partial_{\beta }e_{\alpha }^{i}).$$

The contortion tensor is generated based on the torsion components, as:


$$K_{\alpha \beta }^{\delta }=-\frac{37}{2}\left(T_{\alpha \beta }^{\delta }-T_{\beta \alpha }^{\delta }-T_{\alpha \beta }^{\delta }\right).$$

The torsion scalar is obtained using the spin tensor, which is represented as:

$$S_{\alpha \beta }^{\delta }=\frac{37}{2}\left(K_{\alpha \beta }^{\delta }+\gamma_{\alpha }^{\eta }T_{\xi }^{\xi \beta }-\gamma_{\beta }^{\eta }T_{\xi }^{\xi \beta }\right).$$ (13)

Field equations in *f*(*T*) gravity, can be expressed as:

$$S_{\beta }^{\eta \rho }\partial_{\rho }Tf_{TT}+e^{-1}e_{\xi }^{i}\partial_{\rho }\left(ee_{i}^{k}S_{k}^{\eta \rho }\right)+T_{\phi \xi }^{k}S_{k}^{\eta \phi }f_{T}+\frac{37}{4}\gamma_{\xi }^{\eta }f=4\pi T_{\xi }^{\eta }.$$ (14)

Here f=f(T) and *f*_*T*_, *f*_*TT*_ show the regarding *T*, while *e* indicates the tetrad field’s determinant. Moreover, the torsion scalar *T* is formulated via the contraction below :

$$T=T_{\xi \varphi }^{\eta }S_{\eta }^{\xi \varphi }.$$ (15)

For the spacetime being considered, the tetrad is taken as:


$$e_{a}^{i}=\text{diag}(1,K_{1}(t),K_{2}(t),K_{2}(t)).$$

The non-vanishing components of the superpotential and torsion tensor, turned out:

$$S_{x}^{xt}=\frac{K_{2}^{\prime }}{K_{2}},\;S_{y}^{yt}=S_{z}^{zt}=\frac{37}{2}\left(\frac{K_{1}^{\prime }}{K_{1}}+\frac{K_{2}^{\prime }}{K_{2}}\right).$$ (16)

$$T_{tx}^{x}=\frac{K_{1}^{\prime }}{K_{1}},\;T_{ty}^{y}=T_{tz}^{z}=\frac{K_{2}^{\prime }}{K_{2}},$$ (17)

Eq (15), yields the torsion scalar *T*, as follow:

$$T=-2\left(2\frac{K_{1}^{\prime }K_{2}^{\prime }}{K_{1}k_{2}}+\frac{{K_{2}^{\prime }}^{2}}{K_{2}^{2}}\right).$$ (18)

By modeling the energy-momentum tensor with a perfect fluid, along with the above data, Eq (14) provides the following equations for the spacetime given in Eq (3):

$$f+4\left(2\frac{K_{1}^{\prime }K_{2}^{\prime }}{K_{1}K_{2}}+\frac{{K_{2}^{\prime }}^{2}}{K_{2}^{2}}\right)f_{T}(T)=-16\pi \rho ,$$ (19)

$$f+4\left(\frac{{K_{2}^{\prime }}^{2}}{K_{2}^{2}}+\frac{{K_{2}^{\prime }}^{\prime }}{K_{2}}+\frac{K_{1}^{\prime }K_{2}^{\prime }}{K_{1}K_{2}}\right)f_{T}(T)+4T^{\prime }\frac{K_{2}^{\prime }}{K_{2}}f_{TT}(T)=16\pi p,$$ (20)

$$f+2\left(\frac{{K_{2}^{\prime }}^{2}}{K_{2}^{2}}+\frac{{K_{1}^{\prime }}^{\prime }}{K_{1}}+\frac{{K_{2}^{\prime }}^{\prime }}{K_{2}}+3\frac{K_{1}^{\prime }K_{2}^{\prime }}{K_{1}K_{2}}\right)f_{T}(T)+2T^{\prime }\left(\frac{K_{2}^{\prime }}{K_{2}}+\frac{K_{1}^{\prime }}{K_{1}}\right)f_{TT}(T)=16\pi p.$$ (21)

Where ρ and *p* stand for energy density and pressure respectively. Following some mathematical manipulation on the aforementioned equations, which leads us to the derived single equation:

$$\left(\frac{K_{1}^{\prime }K_{2}^{\prime }}{K_{1}K_{2}}-\frac{{K_{2}^{\prime }}^{2}}{K_{2}^{2}}+\frac{{K_{1}^{\prime }}^{\prime }}{K_{1}}-\frac{{K_{2}^{\prime }}^{\prime }}{K_{2}}\right)f_{T}(T)+\left(\frac{K_{1}^{\prime }}{K_{1}}-\frac{K_{2}^{\prime }}{K_{2}}\right)T^{\prime }f_{TT}(T)=0.$$ (22)

**Fig 1:**
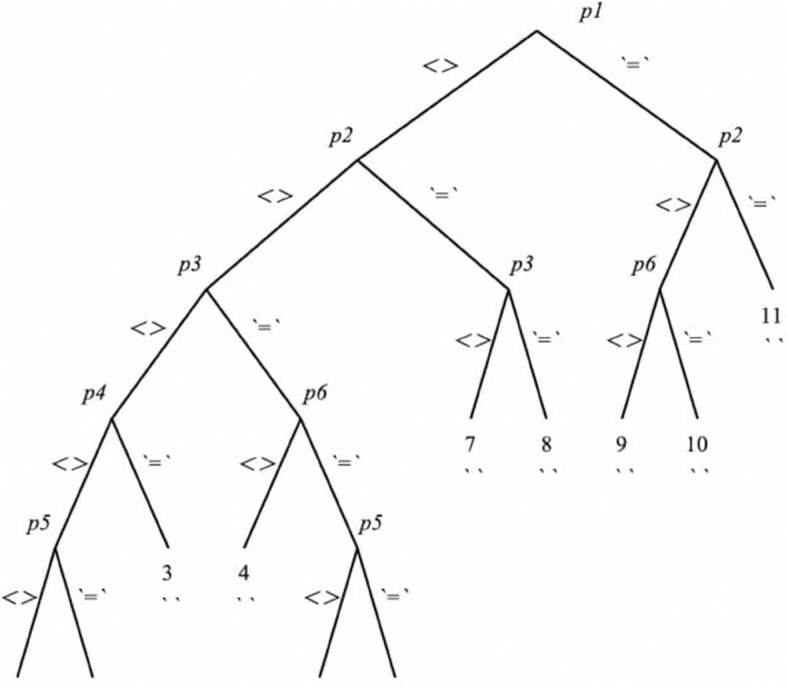
Rif Tree.


For the solution of Eqs (7)–(12) and Eqs (19)–(21) or (22), certain assumptions on *K*_1_ and *K*_2_ are required. To this end, a computer algorithm using **Maple** is employed to analyze these equations. Consequently, the algorithm imposes restrictions on *K*_1_ and *K*_2_ in the form of a tree structure called the **Rif tree**, shown in Fig 1. The tree consists of multiple branches, several pivots (23), and indicators  =  and <>, which specify whether the corresponding pivot is taken to be zero or nonzero. The integration of Eqs (7)–(12) and Eqs (19)–(21) or (22), subject to the constraints of each branch of the tree, leads to the final form of the **SSVFs**, *f*(*T*), *K*_1_, *K*_2_, ρ, and *p*.

$$p1=K_{2}^{\prime },$$

$$p2=K_{1}^{\prime },$$

$$p3=K_{2}{K_{2}^{\prime }}^{\prime }-{K_{2}^{\prime }}^{2},$$

$$p4=K_{1}^{\prime }K_{2}+K_{1}K_{2}^{\prime },$$

$$p5=K_{1}^{\prime }K_{2}-K_{1}K_{2}^{\prime },$$

$$p6={K_{1}^{\prime }}^{2}-K_{1}{K_{1}^{\prime }}^{\prime }.$$ (23)

The details of each branch are given in Tables 1–9.

**Table 1:** Metrics admitting SSVFs.

No./Branch	Metric	SSVF
1a.	$K_{1}=(a_{1}t+\alpha_{1}{)}^{\frac{a_{2}-\alpha_{2}}{a_{1}}},$	$V^{0}=a_{1}t+\alpha_{1},$
(Branch 1)	$K_{2}=(a_{1}t+\alpha_{1}{)}^{\frac{a_{2}-\alpha_{3}}{a_{1}}},$	$V^{1}=a_{2}x+\alpha_{4},$
	$a_{2}\neq \alpha_{1},a_{2}\neq \alpha_{3}$	$V^{2}=\alpha_{3}y-\alpha_{5}z+\alpha_{6},$
	$a_{1}\neq 0,\alpha_{1}\neq \alpha_{3}.$	$V^{3}=\alpha_{3}z+\alpha_{5}y+\alpha_{7}.$
1b.	$K_{1}^{\prime }\neq 0,$	$V^{0}=0,$
(Branch 1)	$K_{2}^{\prime }\neq 0.$	$V^{1}=a_{2}x+\alpha_{1},$
	$K_{2}{K_{2}^{\prime }}^{\prime }-{K_{2}^{\prime }}^{2}\neq 0,$	$V^{2}=a_{2}y-\alpha_{2}z+\alpha_{3},$
	$K_{1}\neq K_{2}.$	$V^{3}=a_{2}z+\alpha_{2}y+\alpha_{4}.$

**Table 2:** Metrics admitting SSVFs.

No./Branch	Metric	SSVF
2a.	$K_{1}=(a_{1}t+\alpha_{1}{)}^{\frac{a_{2}-\alpha_{2}}{a_{1}}},$	$V^{0}=a_{1}t+\alpha_{1},$
(Branch 2)	$K_{2}=K_{1},$	$V^{1}=\alpha_{2}x+\alpha_{3}y+\alpha_{4}z+\alpha_{5},$
	$a_{2}\neq \alpha_{2},$	$V^{2}=\alpha_{2}y-\alpha_{3}x-\alpha_{6}z+\alpha_{7},$
	$a_{1}\neq 0.$	$V^{3}=\alpha_{2}z-\alpha_{4}x+\alpha_{6}y+\alpha_{8}.$
2b.	$K_{1}^{\prime }\neq 0,$	$V^{0}=0,$
(Branch 2)	$K_{2}=k_{1},$	$V^{1}=a_{2}x+\alpha_{1}y+\alpha_{2}z+\alpha_{3},$
	$K_{1}{K_{1}^{\prime }}^{\prime }-{K_{1}^{\prime }}^{2}\neq 0.$	$V^{2}=a_{2}y-\alpha_{1}x-\alpha_{4}z+\alpha_{5},$
		$V^{3}=a_{2}z-\alpha_{2}x+\alpha_{4}y+\alpha_{6}.$

**Table 3:** Metrics admitting SSVFs.

No./Branch	Metric	SSVF
3a.	$K_{1}^{\prime }\neq 0,$	$V^{0}=0,$
(Branch 3)	$K_{1}=\frac{37}{K_{2}},$	$V^{1}=a_{2}x+\alpha_{1},$
	$K_{2}{K_{2}^{\prime }}^{\prime }-{K_{2}^{\prime }}^{2}\neq 0.$	$V^{2}=a_{2}y-\alpha_{2}z+\alpha_{3},$
		$V^{3}=a_{2}z+\alpha_{2}y+\alpha_{4}.$
3b.	$K_{2}=(a_{1}t+\alpha_{1}{)}^{\frac{a_{2}-\alpha_{2}}{a_{1}}},$	$V^{0}=a_{1}t+\alpha_{1},$
(Branch 3)	$K_{1}=\frac{37}{K_{2}},$	$V^{1}=(2a_{2}x-\alpha_{2})x+\alpha_{3},$
	$a_{2}\neq \alpha_{2},$	$V^{2}=\alpha_{2}y-\alpha_{4}z+\alpha_{5},$
	$a_{1}\neq 0.$	$V^{3}=\alpha_{2}z+\alpha_{4}y+\alpha_{6}.$
4a.	$K_{1}^{\prime }\neq 0,$	$V^{0}=0,$
(Branch 4)	$K_{2}\neq k_{1},$	$V^{1}=a_{2}x+\alpha_{2},$
	$K_{2}=e^{\alpha_{1}t},$	$V^{2}=a_{2}y-\alpha_{3}z+\alpha_{4},$
	$\alpha_{1}\neq 0$	$V^{3}=a_{2}z+\alpha_{3}y+\alpha_{5}.$
5a.	$K_{1}=e^{\alpha_{1}t},$	$V^{0}=\alpha_{3},$
(Branch 5)	$K_{2}=e^{\alpha_{2}t},$	$V^{1}=(a_{2}-\alpha_{1}\alpha_{3})x+\alpha_{4},$
	$\alpha_{1}\neq \alpha_{2}\neq 0.$	$V^{2}=(a_{2}-\alpha_{2}\alpha_{3})y-\alpha_{5}z+\alpha_{6},$
		$V^{3}=(a_{2}-\alpha_{2}\alpha_{3})z+\alpha_{5}y+\alpha_{7}.$
6a.	$K_{1}=e^{\alpha_{1}t},$	$V^{0}=\alpha_{2},$
(Branch 6)	$K_{2}=k_{1},$	$V^{1}=(a_{2}-\alpha_{1}\alpha_{2})x+\alpha_{3}y+\alpha_{4}z+\alpha_{5},$
	$\alpha_{1}\neq 0.$	$V^{2}=(a_{2}-\alpha_{1}\alpha_{2})y-\alpha_{3}x-\alpha_{6}z+\alpha_{7},$
		$V^{3}=(a_{2}-\alpha_{1}\alpha_{2})z-\alpha_{4}x+\alpha_{6}y+\alpha_{8}.$
7a.	$K_{1}=\alpha_{1}\neq 0,$	$V^{0}=a_{1}t+\alpha_{2},$
(Branch 7)	$K_{2}=(a_{1}t+\alpha_{2}{)}^{\frac{a_{2}-\alpha_{3}}{a_{1}}},$	$V^{1}=a_{2}x+\alpha_{4},$
	$\alpha_{1}\neq 0.$	$V^{2}=\alpha_{3}y-\alpha_{5}z+\alpha_{6},$
		$V^{3}=\alpha_{3}z+\alpha_{5}y+\alpha_{7}.$

**Table 4:** Metrics admitting SSVFs.

No./Branch	Metric	SSVF
7b.	$K_{1}=\alpha_{1}\neq 0,$	$V^{0}=0,$
(Branch 7)	$K_{2}^{\prime }\neq 0,$	$V^{1}=a_{2}x+\alpha_{2},$
	$K_{2}{K_{2}^{\prime }}^{\prime }-{K_{2}^{\prime }}^{2}\neq 0.$	$V^{2}=a_{2}y-\alpha_{3}z+\alpha_{4},$
		$V^{3}=a_{2}z+\alpha_{3}y+\alpha_{5}.$
8a.	$K_{1}=\alpha_{1}\neq 0,$	$V^{0}=\alpha_{3},$
(Branch 8)	$K_{2}=e^{\alpha_{2}},$	$V^{1}=a_{2}x+\alpha_{4},$
	$\alpha_{2}\neq 0.$	$V^{2}=(a_{2}-\alpha_{2}\alpha_{3})y-\alpha_{5}z+\alpha_{6},$
		$V^{3}=(a_{2}-\alpha_{2}\alpha_{3})z+\alpha_{5}y+\alpha_{7}.$
9a.	$K_{1}=(a_{1}t+\alpha_{1}{)}^{\frac{a_{2}-\alpha_{2}}{a_{1}}},$	$V^{0}=a_{1}t+\alpha_{1},$
(Branch 9)	$K_{2}=\alpha_{3}\neq 0,$	$V^{1}=\alpha_{2}x+\alpha_{4},$
	$\alpha_{2}\neq a_{2}.$	$V^{2}=a_{2}y-\alpha_{5}z+\alpha_{6},$
		$V^{3}=a_{2}z+\alpha_{5}y+\alpha_{7}.$
9b.	$K_{1}^{\prime }\neq 0,$	$V^{0}=0,$
(Branch 9)	$K_{2}=\alpha_{1}\neq 0,$	$V^{1}=a_{2}x+\alpha_{2},$
	${K_{1}^{\prime }}^{\prime }-K_{1}{K_{1}^{\prime }}^{2}\neq 0.$	$V^{2}=a_{2}y-\alpha_{3}z+\alpha_{4},$
		$V^{3}=a_{2}z+\alpha_{3}y+\alpha_{5}.$
10a.	$K_{1}=e^{\alpha_{1}t},$	$V^{0}=\alpha_{3},$
(Branch 10)	$K_{2}=\alpha_{2}\neq 0,$	$V^{1}=(a_{2}-\alpha_{1}\alpha_{3})x+\alpha_{4},$
	$\alpha_{1}\neq 0.$	$V^{2}=a_{2}y-\alpha_{5}z+\alpha_{6},$
		$V^{3}=a_{2}z+\alpha_{5}y+\alpha_{7}.$
11a.	$K_{1}=\alpha_{1}\neq 0,$	$V^{0}=a_{1}t+\alpha_{3},$
(Branch 11)	$K_{2}=\alpha_{2}\neq 0.$	$V^{1}=a_{2}x+\alpha_{4}y-\frac{\alpha_{2}^{2}}{\alpha_{1}^{2}}\alpha_{5}z+\alpha_{6},$
		$V^{2}=a_{2}y-\frac{\alpha_{1}^{2}}{\alpha_{2}^{2}}\alpha_{4}x-\alpha_{7}z+\alpha_{8},$
		$V^{3}=a_{2}z+\alpha_{5}x+\alpha_{7}y+\alpha_{9}.$

**Table 5:** Values of ρ,p,T,f(t)s.

Branch No.	Values if ρ, p.	*T*	*f(T)*	Kind of SSVF
4	p=const.=−ρ	$T=-2\alpha_{1}^{2}$	f(T)=const.	Zeroth kind.
5	p=const.=−ρ	$T=-2(2\alpha_{1}\alpha_{2}+\alpha_{2}^{2})$	f(T)=const.	Zeroth kind.
6	p=const.=−ρ	$T=-62\alpha_{2}^{2}$	f(T)=const.	Zeroth kind.
7b	p=const.=−ρ	$T=-2\alpha_{1}^{2}$	f(T)=const.	Zeroth kind
8	$p=\frac{const.}{16\pi }=-\rho$	*T* = 0	f(T)=const.	Zeroth kind.
9	$p=\frac{const.}{16\pi }=-\rho$	*T* = 0	f(T)=const.	First, second and infinite kind.
10	$p=\frac{const.}{16\pi }=-\rho$	*T* = 0	f(T)=const.	Zeroth kind.
11	p=const.=−ρ	*T* = 0	f(T)=const.	First, second and infinite kind.

**Table 6:** Values of ρ,p,T,f(t).

Branch	Values of ρ, p.	Kind of SSVF
3a.	$\rho =-\frac{37}{16\pi }\left(f(T)-4(a_{2}-\alpha_{2}{)}^{2}(a_{1}t+\alpha_{1}{)}^{-2}f_{T}(T)\right).$	First, Second
	$p=\frac{37}{16\pi }\left(f(T)+4(a_{2}-\alpha_{2})(a_{2}-\alpha_{2}-a_{1})(a_{1}t+\alpha_{1}{)}^{-2}f_{T}(T)\right)$	and infinite.
	$+\frac{37}{16\pi }\left(4\dot{T}(a_{2}-\alpha_{2})(a_{1}t+\alpha_{1}{)}^{-1}f_{TT}(T)\right)$	
	Term *T*	*f*(*T*)
	$T=2(a_{2}-\alpha_{2}{)}^{2}(a_{1}t+\alpha_{1}{)}^{-2}$	If$\frac{a_{2}-\alpha_{2}-a_{1}}{2a_{1}}\neq -1$
		then $f(T)=C_{1}\frac{T^{\frac{a_{2}-\alpha_{2}-a_{1}}{2a_{1}}+1}}{\frac{a_{2}-\alpha_{2}-a_{1}}{2a_{1}}+1}+C_{2}$
		if $\frac{a_{2}-\alpha_{2}-a_{1}}{2a_{1}}=-1$
		then $f(T)=C_{1}\ln T+C_{2}$

**Table 7:** Values of ρ,p,T,f(t).

Branch	Values of ρ, p.	Kind of SSVF
7a.	$\rho =-\frac{37}{16\pi }\left(f+4(a_{2}-\alpha_{3}{)}^{2}(a_{1}t+\alpha_{2}{)}^{-2}f_{T}(T)\right)$	First, Second
	$p=\frac{37}{16\pi }\left(f+K(a_{1}t+\alpha_{2}{)}^{-2}f_{T}(T)+4\dot{T}\frac{f_{2}^{\prime }}{f_{2}}f_{TT}(T)\right)$	and infinite.
	where $K=4\left((a_{2}-\alpha_{3}{)}^{2}+(a_{2}-\alpha_{3})(a_{2}-\alpha_{3}-a_{1})\right)$	
	Term *T*	f(T)
	$T=-2(a_{2}-\alpha_{3}{)}^{2}(a_{1}t+\alpha_{2}{)}^{-2}$	$f(T)=C\frac{T^{1-\frac{k}{a_{1}t+\alpha_{2}}}}{1-\frac{k}{a_{1}t+\alpha_{2}}}+D$
		where $k=2(a_{2}-\alpha_{3})-a_{1}$

**Table 8:** Kinematic variables (Θ, $\sigma_{11}$, $\sigma_{22}$) for Branches 1a–7a in Bianchi type–I spacetimes under *f*(*T*) gravity. Expressions depend on parameters *a*_*i*_, $\alpha_{i}$, and function *K*_1_(*t*).

No./Branch	Kinematic variables
1a.	$\Theta =\frac{6a_{2}-2\alpha_{2}-4\alpha_{3}}{a_{1}t+\alpha_{1}},$
(Branch 1)	$\sigma_{11}=2(a_{2}-\alpha_{2})(a_{1}t+\alpha_{1}{)}^{2\frac{a_{2}-\alpha_{2}}{a_{1}}-1},$
	$\sigma_{22}=2(a_{2}-\alpha_{3})(a_{1}t+\alpha_{1}{)}^{2\frac{a_{2}-\alpha_{3}}{a_{1}}-1}$.
2a.	$\Theta =\frac{6(a_{2}-\alpha_{2})}{a_{1}t+\alpha_{1}},$
(Branch 2)	$\sigma_{11}=2(a_{2}-\alpha_{2})(a_{1}t+\alpha_{1}{)}^{2\frac{a_{2}-\alpha_{2}}{a_{1}}-1},$
	$\sigma_{22}=\sigma_{11}$.
2b.	$\Theta =\frac{6K_{1}^{\prime }}{K_{1}},$
(Branch 2)	$\sigma_{11}=2K_{1}K_{1}^{\prime },$
	$\sigma_{22}=\sigma_{11}$.
4a.	$\Theta =\frac{2K_{1}^{\prime }}{K_{1}}+4\alpha_{1},$
(Branch 4)	$\sigma_{11}=2K_{1}k_{1}^{\prime },$
	$\sigma_{22}=2\alpha_{1}e^{2\alpha_{1}t}$.
5a.	$\Theta =2(\alpha_{1}+2\alpha_{2}),$
(Branch 5)	$\sigma_{11}=2\alpha_{1}e^{2\alpha_{1}t},$
	$\sigma_{22}=2\alpha_{2}e^{2\alpha_{2}t}$.
6a.	$\Theta =6\alpha_{1},$
(Branch 6)	$\sigma_{11}=2\alpha_{1}e^{2\alpha_{1}t},$
	$\sigma_{22}=\sigma_{11}$.
7a.	$\Theta =\frac{4(a_{2}-\alpha_{3})}{a_{1}t+\alpha_{2}},$
(Branch 7)	$\sigma_{11}=0,$
	$\sigma_{22}=2(a_{2}-\alpha_{3})(a_{1}t+\alpha_{1}{)}^{2\frac{a_{2}-\alpha_{3}}{a_{1}}-1}$.

**Table 9:** Kinematic variables (Θ, $\sigma_{11}$, $\sigma_{22}$) for Branches 7b–11a, including vanishing shear cases. Dependencies on *a*_*i*_, $\alpha_{i}$, *K*_1_(*t*), and *K*_2_(*t*) are shown.

No./Branch	Kinematic variables
7b.	$\Theta =\frac{4K_{2}^{\prime }}{K_{2}},$
(Branch 7)	$\sigma_{11}=0,$
	$\sigma_{22}=2K_{2}K_{2}^{\prime }$.
8a.	$\Theta =4\alpha_{2},$
(Branch 8)	$\sigma_{11}=0,$
	$\sigma_{22}=2\alpha_{2}e^{2\alpha_{2}t}$.
9a.	$\Theta =\frac{2(a_{2}-\alpha_{2})}{a_{1}t+\alpha_{1}},$
(Branch 9)	$\sigma_{11}=2(a_{2}-\alpha_{2})(a_{1}t+\alpha_{1}{)}^{2\frac{a_{2}-\alpha_{2}}{a_{1}}-1},$
	$\sigma_{22}=0$.
9b.	$\Theta =\frac{2K_{1}^{\prime }}{K_{1}},$
(Branch 9)	$\sigma_{11}=2K_{1}K_{1}^{\prime },$
	$\sigma_{22}=0$.
10a.	$\Theta =2\alpha_{1},$
(Branch 9)	$\sigma_{11}=2\alpha_{1}e^{2\alpha_{1}t},$
	$\sigma_{22}=0$.
11a.	Θ=0,
(Branch 10)	$\sigma_{11}=0,$
	$\sigma_{22}=0$.

## 3 Main results

The primary outcomes of this research are detailed in this section, highlighting the key findings and their implications for the broader understanding of the spacetime under study.

### 3.1 Branch-1

The integration of Eqs (7)–(12) for branch 1 leads to the results given below:

Here, both the constants *a*_1_ and *a*_2_ turn out to be nonzero. Thus, the obtained SSVFs correspond either to the first kind or the second kind in metric 1a. Moreover, in metric 2a, the condition *a*_1_ = 0 and $a_{2}\neq 0$ ensures that the given spacetime admits zeroth-kind SSVFs.

To discuss pressure and density, let


$$n_{1}=\frac{a_{2}-\alpha_{2}}{a_{1}},\;n_{2}=\frac{a_{2}-\alpha_{3}}{a_{1}}.$$

Then, one may show that the torsion scalar is obtained as:


$$T=-2\frac{(2n_{1}n_{2}+n_{2}^{2})a_{1}^{2}}{(a_{1}t+\alpha_{1}{)}^{2}}.$$

Defining


$$D=2n_{1}n_{2}+n_{2}^{2},\;\frac{a_{1}^{2}}{(a_{1}t+\alpha_{1}{)}^{2}}=-\frac{T}{2D},$$

the field equations reduce to:

$$f-2T\;f_{T}=-16\pi \;\rho ,$$ (24)

$$f-\frac{2T\;[2n_{2}^{2}+n_{2}(n_{1}-1)]}{D}\;f_{T}+4T\;n_{2}\sqrt{-\frac{T}{2D}}\;f_{TT}=16\pi \;p.$$ (25)

The complex mathematical structure of the above equations makes it difficult to obtain exact values of ρ, *f*(*T*), and *p*. To address this, a common choice in *f*(*T*) gravity, the power-law form $f(T)=f_{0}\;T^{m}$, is used in the system above, yielding:

$$\rho =-\frac{f_{0}(1-2m)}{16\pi }\;T^{m},$$ (26)

$$p=\frac{f_{0}}{16\pi }\left\{T^{m}\left[1-\frac{2m\;(2n_{2}^{2}+n_{2}(n_{1}-1))}{D}\right]+4\;m(m-1)n_{2}\;T^{m-1}\sqrt{-\frac{T}{2D}}\right\}.$$ (27)

For the particular choices *f*_0_ = 1, *m* = 2, $n_{1}=n_{2}=1$, the expressions for energy density and pressure simplify to:


$$\rho (T)=\frac{3}{16\pi }\;T^{2},$$

$$p(T)=\frac{37}{16\pi }\left[-\frac{5}{3}\;T^{2}+8\;T\;\sqrt{-\frac{T}{6}}\right].$$

**Fig 2:**
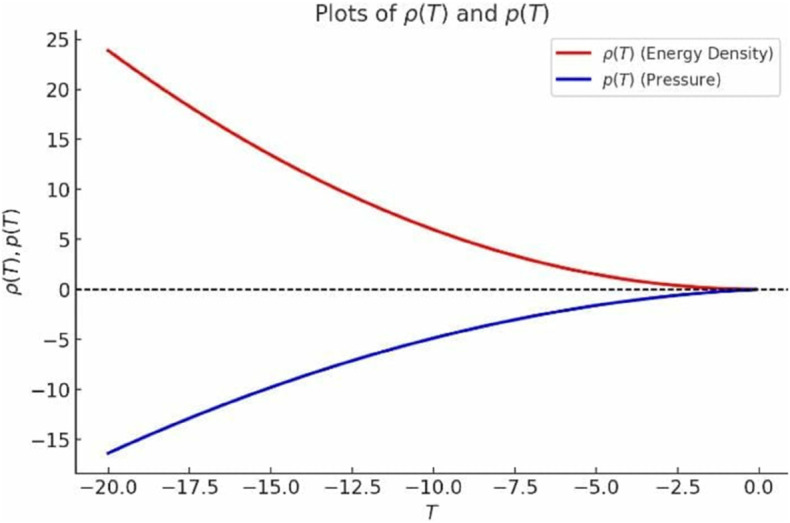
Graph of *ρ* and p for branch 1.

Since *T* < 0 (as required by the factor $\sqrt{-T/6}$), we plot these functions for T∈[−20,−0.1], as shown in Fig 2. Since *T*^2^ > 0 for T≠0, we have ρ(T)>0. As |T| increases (i.e., moving left along the negative *T*-axis from *T* = −0.1 to *T* = −20), *T*^2^ increases, leading to an increase in ρ(T). Conversely, as *T* moves from –20 toward –0.1, *T*^2^ decreases, causing ρ(T) to decrease.

Furthermore, the first term $\left(-\frac{5}{3}T^{2}\right)$ and the second term $8T\sqrt{-\frac{T}{6}}$ are always negative. Since both terms remain negative, *p*(*T*) is negative for all *T*<0. This indicates that *p*(*T*) is negative throughout the given range, but its magnitude decreases, meaning it becomes less negative as *T* increases from –20 to –0.1.

### 3.2 Branch 2

The symmetry equations yield the following outcomes under the constraints of branch 2. In metric 2*a*, SSVFs are either of the first or second kind, while in 2*b*, the condition *a*_1_ = 0 and $a_{2}\neq 0$ demonstrates that the LRS Bianchi-I spacetime possesses SSVFs of the zeroth kind.

The equality of both functions $K_{1}=K_{2}$ in this branch provides


$$T=-6\frac{n^{2}}{(a_{1}t+\alpha_{1}{)}^{2}},$$

where $n=\frac{a_{2}-\alpha_{2}}{a_{1}}$. A similar assumption for the term *f*(*T*) in the aforementioned field equations yields:


$$\rho =-\frac{f_{0}(1-2m)}{16\pi }\;T^{m},$$

$$p=\frac{f_{0}}{16\pi }\left[T^{m}-\frac{2m(3n-1)}{3n}\;T^{m}+4m(m-1)T^{m-1}\sqrt{-\frac{T}{6}}\right].$$

Taking specific values *f*_0_ = 1, *m* = 2, and *n* = 1, with 16π≈50.24, the expressions simplify to:


$$\rho =-\frac{\left(1-2(2)\right)}{50.24}T^{2}=\frac{T^{2}}{50.24},$$

which is always non-negative. Similarly, the pressure equation becomes:


$$p=\frac{37}{50.24}\left[T^{2}-\frac{2(2)(3(1)-1)}{3(1)}T^{2}+4(2)(2-1)T\sqrt{-\frac{T}{6}}\right].$$

**Fig 3:**
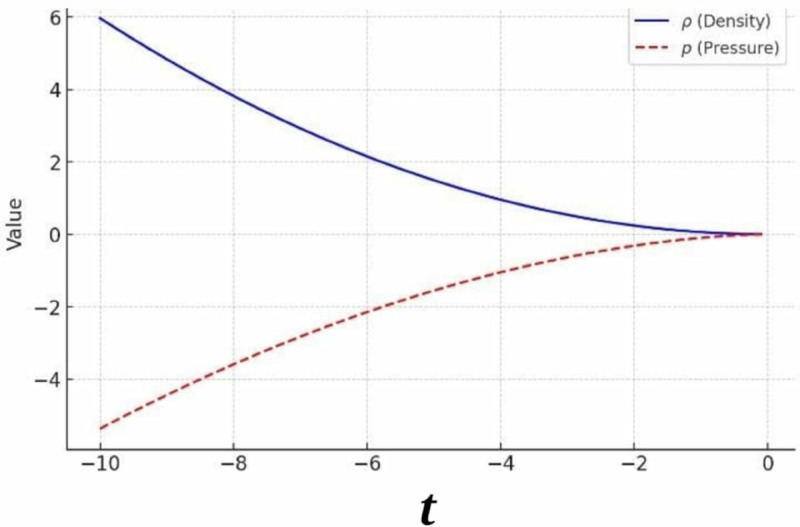
Graph of *ρ* and p for branch 2.

The term $\sqrt{\left|T/6\right|}$ ensures real values for all *T*, requiring *T* to be nonzero. For large negative values of *T*, both ρ and *p* increase in magnitude, indicating significant variations in their behavior, as shown in Fig 3.

The positive density suggests standard thermodynamic behavior, while deviations in pressure could provide insights into modified physics scenarios. This analysis confirms that the system’s properties are highly sensitive to variations in *T*, with both pressure and density exhibiting a strong dependence on its value under the given conditions. For the remaining branches, the solutions of the symmetry equations and the field equation Eq (22) have been carried out under the corresponding branch constraints. Tables 3 and 4 contain the particular values of $f_{1},f_{2}$, along with the symmetry vector fields obtained for each branch of the tree. The specific forms of *f*(*T*), ρ, *p*, *T*, and the associated kinds of SSVFs are presented in Tables 5–7.

Fixing the constants such that $a_{1}=1,\;a_{2}=2,\;\alpha_{1}=1,\;\alpha_{2}=0.5,\;C_{1}=1,\;C_{2}=0$ and t∈[0.1 10], the graph of *ρ* and *p* is plotted as:

The value of *a*_1_ turns out to be zero in branch 3 (metric a), 4-6, 7 (metric b), 8, 9 (metric b), and 10, while *a*_2_ may take any value. If *a*_2_ = 0, then the SSVFs reduce to Killing symmetries of different dimensions, including 5, 6, and 7. Moreover, if we keep *a*_2_ nonzero, then the SSVFs of all the mentioned branches turn out to be of the zeroth kind.

In the remaining branches, namely 3 (metric b), 7 (metric a), 9 (metric a), and 11, the constant *a*_2_ is found to be arbitrary, while $a_{1}\neq 0$. Under these conditions, the spacetime admits SSVFs under the following three possibilities regarding the constants:

(i). If $a_{1}=a_{2}$, the spacetime admits SSVFs of the first kind.
(ii). SSVFs are of the second kind when $a_{1}\neq a_{2}\neq 0,1$.
(iii). When *a*_2_ = 0, the SSVFs reduce to the infinite kind.

**Fig 4:**
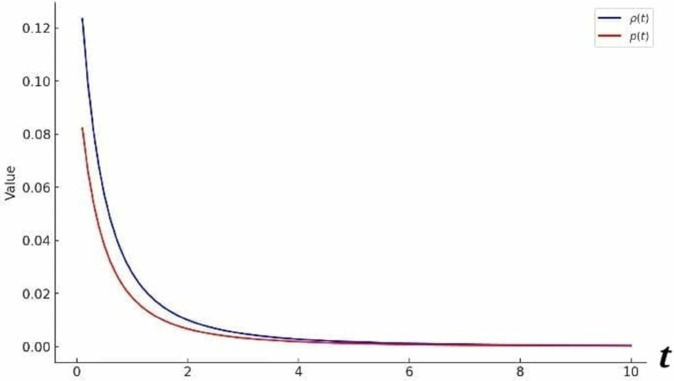
Graph of *ρ* and p for metric 3a.

Fig 4 shows that the energy density ρ and pressure *p* both decrease over time. This behavior suggests an expanding universe where energy density dilutes and pressure evolves accordingly. The different rates of decrease indicate a varying equation of state, potentially resembling dark energy if *p* becomes negative. The smooth decline without oscillations suggests a stable and consistent cosmological evolution.

**Fig 5:**
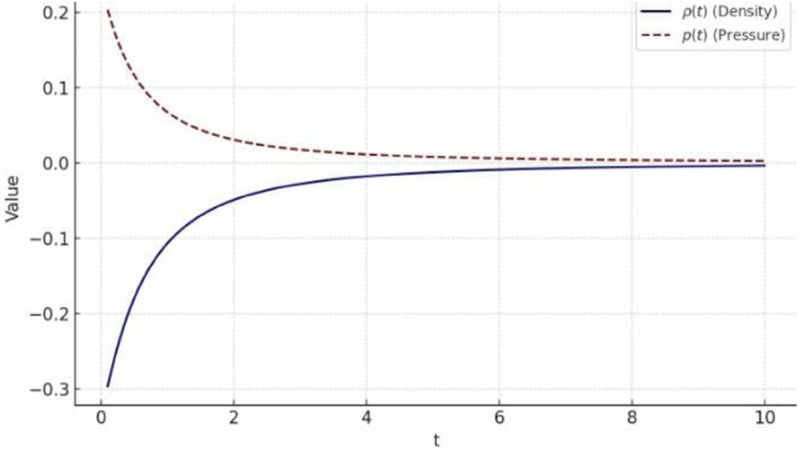
graph of *ρ* and p for metric 7a.

Under the particular values of the constants involved in *ρ* and *p*, that is $a_{1}=1,\;a_{2}=2,\;\alpha_{2}=1,\;\alpha_{3}=0.5,\;C_{1}=1,\;C_{2}=0,$ and t∈[0.1 10], the graph given in Fig 5 is obtained:

The graph illustrates the evolution of energy density *ρ* and pressure *p*. The energy density *ρ* decreases as *t* increases, indicating a dilution effect, which is consistent with cosmic expansion. Similarly, the pressure *p* also declines, with possible negative values suggesting an accelerating expansion phase.

## 4 Kinematic variables

This section deals with the kinematic variables, including acceleration, expansion, shear, and rotation for the LRS Bianchi-I spacetime. These terms are defined as [7]:

1. Acceleration → $\dot{u_{i}}=u_{i;j}u^{j}$.
2. Expansion → $\Theta =u_{;i}^{i}.$
3. Shear → $\sigma_{ij}=u_{\left(i;j\right)}+{\dot{u}}_{[i}u_{j]}-\frac{37}{3}\Theta h_{ij}.$
4. Rotation → $\omega_{ij}=u_{\left[i;j\right]}+{\dot{u}}_{[i}u_{j]}$.

Moreover, the term $\sigma_{ij}$ describes the deformation occurring in fluid flow while keeping the fluid volume invariant, whereas Θ is used to analyze volume behavior. For the LRS Bianchi-I spacetime, we have $u=(u_{0},u_{1},u_{2},u_{3})=(1,0,0,0)$. By substituting *u* and the metric (3) into the above kinematic variable expressions, we obtain the following nonzero components:

$$\dot{u_{i}}=0,\;\omega_{ij}=0,\;\Theta =\frac{2K_{1}^{\prime }}{K_{1}}+\frac{4K_{2}^{\prime }}{k_{2}},\;\sigma_{11}=2K_{1}K_{1}^{\prime },\;\sigma_{22}=\sigma_{33}=2K_{2}K_{2}^{\prime }.$$ (28)

Tables 8 and 9 capture the specific values of all the above terms for all the branches of the tree.

## 5 Comparison

This section is concerned with the comparison comments of the said paper with [48]. The main focus of this article is to classify the LRS Bianchi type-I spacetime via self-similar symmetry, whereas [48] focused on studying the same spacetime using Ricci soliton vector fields. This article adopts the Riftree approach, while [48] uses the direct integration approach. In our article, we have derived explicit values for the spacetime metric functions, whereas in [48], the authors mostly assume specific values for these functions rather than deriving them. Additionally, we have analyzed the graphical behavior of energy density and pressure, which is not included in [48]. These qualities, set our paper apart make it truly exceptional compared to the one it’s being compared with.

## 6 Conclusion

In the context of *f*(*T*) gravity, our primary goal was to explore and identify SSVFs in the spacetime under study, particularly in the presence of a perfect fluid. To systematically address this problem, we divided our work into four parts. First, we derived the SSVF equations. Second, we formulated the field equations for the given spacetime, incorporating the perfect fluid, and transformed these field equations along with the symmetry equations using Maple to obtain a set of constraints on the metric functions of the spacetime. This process led to a variety of possible solutions, each of which we carefully examined, categorized into distinct cases. Moreover, for each case, we calculated key physical quantities, including the fluid pressure (*p*), energy density (ρ), torsion scalar (*T*), and the corresponding functional form of *f*(*T*). A kinematic variables for all the derived metrics are also discussed, to highlight the significance of our work. Furthermore, our results demonstrate that the LRS Bianchi-I spacetime admits SSVFs of each kind. Additionally, to analyze the physical viability of our findings, the physical terms ρ and *p* were calculated and plotted graphically. This approach not only highlights the flexibility of our method but also underscores the importance of exploring alternative strategies in theoretical physics research.

Our future work will extend this idea and methodological approach to different modified gravity theories.
